# Genomic Profiling Reveals an Alternate Mechanism for Hepatic Tumor Promotion by Perfluorooctanoic Acid in Rainbow Trout

**DOI:** 10.1289/ehp.11190

**Published:** 2008-05-09

**Authors:** Susan C. Tilton, Gayle A. Orner, Abby D. Benninghoff, Hillary M. Carpenter, Jerry D. Hendricks, Cliff B. Pereira, David E. Williams

**Affiliations:** 1 Department of Environmental and Molecular Toxicology; 2 Marine and Freshwater Biomedical Sciences Center; 3 Linus Pauling Institute; 4 Environmental Health Sciences Center and; 5 Department of Statistics, Oregon State University, Corvallis, Oregon, USA

**Keywords:** clofibrate, dehydroepiandrosterone, estradiol, hepatocarcinogenesis, microarray, perfluorooctanoic acid, peroxisome proliferation, rainbow trout

## Abstract

**Background:**

Perfluorooctanoic acid (PFOA) is a potent hepatocarcinogen and peroxisome proliferator (PP) in rodents. Humans are not susceptible to peroxisome proliferation and are considered refractory to carcinogenesis by PPs. Previous studies with rainbow trout indicate they are also insensitive to peroxisome proliferation by the PP dehydroepiandrosterone (DHEA), but are still susceptible to enhanced hepatocarcinogenesis after chronic exposure.

**Objectives:**

In this study, we used trout as a unique *in vivo* tumor model to study the potential for PFOA carcinogenesis in the absence of peroxisome proliferation compared with the structurally diverse PPs clofibrate (CLOF) and DHEA. Mechanisms of carcinogenesis were identified from hepatic gene expression profiles phenotypically anchored to tumor outcome.

**Methods:**

We fed aflatoxin B_1_ or sham-initiated animals 200–1,800 ppm PFOA in the diet for 30 weeks for tumor analysis. We subsequently examined gene expression by cDNA array in animals fed PFOA, DHEA, CLOF, or 5 ppm 17β-estradiol (E_2_, a known tumor promoter) in the diet for 14 days.

**Results:**

PFOA (1,800 ppm or 50 mg/kg/day) and DHEA treatments resulted in enhanced liver tumor incidence and multiplicity (*p* < 0.0001), whereas CLOF showed no effect. Carcinogenesis was independent of peroxisome proliferation, measured by lack of peroxisomal β-oxidation and catalase activity. Alternately, both tumor promoters, PFOA and DHEA, resulted in estrogenic gene signatures with strong correlation to E_2_ by Pearson correlation (*R* = 0.81 and 0.78, respectively), whereas CLOF regulated no genes in common with E_2_.

**Conclusions:**

These data suggest that the tumor-promoting activities of PFOA in trout are due to novel mechanisms involving estrogenic signaling and are independent of peroxisome proliferation.

Perfluorooctanoic acid (PFOA) is a member of a class of perfluorinated compounds that are used widely in consumer products and industrial applications including surfactants, lubricants, textile coatings, food packaging, and flame retardants. PFOA is also a degradation product of other fluoropolymers that is highly resistant to further metabolic and environmental breakdown. Because of its widespread occurrence and chemical stability, there are increasing concerns about the environmental persistence and accumulation of PFOA measured in terrestrial and aquatic biota and in human serum (Calafat et al. 2007; Houde et al. 2006; Prevedouros et al. 2006). Estimation of PFOA half-lives in serum varies broadly, depending on species and sex, and ranges from days in rats (Vanden Heuvel et al. 1991) and cynomolgus monkeys (Butenhoff et al. 2004) to almost 4 years in occupationally exposed humans (Olsen et al. 2007). Although some perfluorinated chemicals have been voluntarily removed from the market by manufacturers over concerns related to environmental occurrence and stability, PFOA is still produced commercially, and its potential risk to humans continues to be evaluated (U.S. Environmental Protection Agency 2006).

PFOA is a potent peroxisome proliferator (PP) similar to other perfluorinated chemicals (Sohlenius et al. 1992). Overall, PPs comprise a structurally diverse group of nongenotoxic carcinogens including certain hypolipidemic drugs [clofibrate (CLOF), ciprofibrate], industrial plasticizers (phthalates), herbicides (phenoxyacetic acids), and organic solvents (trichloroethylene). PPs are known to cause hepatomegaly, altered cholesterol homeostasis, increased number and size of peroxisomes, and increased β-oxidation and ω-oxidation of fatty acids in peroxisomes and microsomes, respectively, in susceptible animal models (Moody et al. 1991). Prolonged exposure to PPs, including PFOA, also results in increased liver tumor incidence in rodents (Abdellatif et al. 1991). Although the causative link between peroxisome proliferation and hepatocarcinogesis has not been firmly established, PPs are thought to enhance liver tumors in rodent models as a result of peroxisome proliferation through sustained oxidative stress and regulation of cellular proliferation and differentiation (Reddy and Rao 1989). Many PP-mediated effects occur through ligand-dependent activation of the nuclear receptor, PP-activated receptor alpha (PPARα), and consequently do not occur in PPARα-null mice (Peters et al. 1997).

Despite the apparent toxicity of PFOA and other PPs in rodents, humans are relatively insensitive to peroxisome proliferation by this class of compounds (Fruchart et al. 1998). In fact, marked species differences exist in susceptibility to peroxisome proliferation such that rodents are highly sensitive, whereas humans and certain other models, including rainbow trout, guinea pigs, and nonhuman primates, show little to no evidence of peroxisome proliferation (Butenhoff et al. 2002; Lake et al. 1989; Orner et al. 1996). The insensitivity of humans to PP toxicity is attributed to the approximately 10-fold lower expression of PPARα receptor in liver compared with mouse (Palmer et al. 1998). It is generally accepted that humans are likely refractory to hepatocarcinogenesis caused by peroxisome proliferation via a PPARα mode of action; however, there is also accumulating evidence for PFOA toxicity independent of peroxisome proliferation. For example, PFOA induces Leydig-cell and pancreatic acinar-cell tumors in chronic rodent bioassays, similar to some other PPs (Biegel et al. 2001). Leydig-cell tumor formation was correlated with elevated serum estradiol and inhibition of testosterone synthesis rather than peroxisome proliferation in these cells. PFOA was also shown to cause hepatomegaly in PPARα-null mice and cynomolgus monkeys without an increase in typical measures of peroxisome proliferation, suggesting that PFOA can cause liver toxicity presumably independent of PPARα, which may be relevant for humans (Butenhoff et al. 2002; Yang et al. 2002). These studies show the potential for significant toxicity by PFOA to occur independent of peroxisome proliferation and indicate that use of PP-insensitive species in evaluation of novel PFOA effects may be important for identification of mechanisms that can be extrapolated to humans.

In this study, we examined the *in vivo* effects of chronic PFOA exposure on hepatocarcinogenesis in rainbow trout, a model used for chemically induced liver cancer in humans for over 40 years (Bailey et al. 1996). PFOA was evaluated as both a complete carcinogen and as a promoter of aflatoxin B_1_ (AFB_1_)-induced liver cancer compared with two structurally diverse PPs, CLOF and dehydroepiandrosterone (DHEA). CLOF is a hypolipidemic drug and known PPARα agonist that acts as a complete carcinogen in rodents (Reddy and Qureshi 1979). DHEA, an adrenal steroid in humans, is classified as a PP based on its ability to induce peroxisome proliferation in rodents, but appears to operate independently of PPARα (Webb et al. 2006). Our laboratory previously demonstrated that rainbow trout are insensitive to peroxisome proliferation by DHEA but are still responsive to induction of liver cancer by this compound (Orner et al. 1995). Rainbow trout are also susceptible to tumor enhancement by pro-oxidants (Kelly et al. 1992), which is a proposed mechanism for PP-induced liver cancer in rodents. The goals of this study were to *a*) determine the potential for PFOA-mediated carcinogenesis *in vivo* in the absence of peroxisome proliferation, *b*) investigate whether this mode of action is common across a class of known PPs or PPARα agonists, and *c*) identify potential mechanisms of carcinogenesis from phenotypic anchoring of global gene expression profiles to tumor outcome. Toxicogenomic profiling has been successfully used in rainbow trout to examine mechanisms of chemical hepatocarcinogenesis (Tilton et al. 2005, 2006, 2007) and has also been used in other models to determine effects of PFOA in liver (Guruge et al. 2006). Overall, we determined that PFOA can enhance hepatocarcinogenesis postinitiation in the trout model at 1,800 ppm in the diet, or 50 mg/kg/day. However, tumor promotion was not correlated to peroxisome proliferation, but rather to estrogenic signaling in trout liver, which is a novel mechanism of carcinogenicity reported for PFOA in a PP-insensitive species.

## Materials and Methods

### Materials and animals

Analytical-grade AFB_1_, 17β-estradiol (E_2_), and DHEA were purchased from Sigma Chemical Co. (St. Louis, MO). CLOF and PFOA were purchased from Fluka Chemical Corp. (St. Louis, MO). All other compounds were purchased from Sigma Chemical Co. unless otherwise stated. Mt. Shasta strain rainbow trout (*Oncorhynchus mykiss*) were hatched and reared at the Oregon State University Sinnhuber Aquatic Research Laboratory in 14°C flowing well water on a 12:12 hr light:dark cycle. All animal protocols were performed in accordance with Oregon State University Institutional Animal Care and Use Committee guidelines. Animals were treated humanely and with regard for alleviation of suffering.

### Tumor experiment, necropsy, and histopathology

Approximately 1,000 fry were initiated at 10 weeks posthatch with an aqueous exposure to 0.01 ppm AFB_1_ for 30 min. Sham-exposed trout were exposed to vehicle alone (0.01% ethanol) and served as non-initiated controls for each treatment. After initiation, fry were fed Oregon Test Diet (OTD), a semipurified casein-based diet, for 3 months (Lee et al. 1991). Trout were then randomly (within initiator group) divided into experimental treatment groups (140 animals/treatment) and fed experimental diets containing 200 or 1,800 ppm PFOA, 1,800 ppm CLOF, or 1,800 ppm DHEA *ad libitum* (2.8–5.6% body weight) 5 days/week for 6 months, a protocol similar to that previously described for DHEA (Orner et al. 1995). The PFOA concentrations in the diet for 200 and 1,800 ppm are equivalent to 5 and 50 mg/kg/day, respectively. Diets were prepared monthly and stored frozen at −20°C until 2–4 days prior to feeding, when diets were allowed to thaw at 4°C.

At 9 months postinitiation, juvenile fish were euthanized by deep anesthesia with 250 ppm tricaine methanesulfonate and sampled for liver tumors over a 2-day period. Livers were fixed in Bouin’s solution for 2–7 days for histologic identification and examination of tumors with hematoxylin and eosin. Neoplasms were classified by the criteria of Hendricks et al. (1984). The effect of dietary PPs on tumor incidence was modeled by logistic regression (GENMOD procedure, SAS version 9.1; SAS Institute, Cary, NC). Tumor multiplicity data (number of tumors per tumor-bearing animal) were analyzed by the Kruskal-Wallis test with *p*-values based on the exact permutation distribution (StatXact, version 2.04; Cytel Software, Corp., Cambridge, MA).

### Microarray experiment

Juvenile trout, 12–18 months of age, were maintained in separate 375-L tanks (three tanks) for each treatment, with five fish per tank. Animals were fed a maintenance ration (2.8% wt/tw) of OTD. Administration of experimental diets containing 500 or 1,800 ppm PFOA, 1,800 ppm CLOF, 750 ppm DHEA, 5 ppm E_2_, or 0.1% dimethyl sulfoxide (DMSO) vehicle control was carried out for 14 days. The concentrations of E_2_ and DHEA were chosen based on their ability to maximally induce vitellogenin (VTG) and/or act as hepatic tumor promoters in trout (Nunez et al. 1989). On day 15, fish were euthanized by deep anesthesia with 250 ppm tricaine methanesulfonate. Approximately 100 mg liver tissue from individual fish was minced, stored in TRIzol Reagent (Invitrogen, Carlsbad, CA) and quick-frozen in liquid nitrogen for gene expression analysis. The rest of the liver was quick-frozen in liquid nitrogen for enzyme assays.

Total hepatic RNA was isolated from individual trout liver using TRIzol Reagent, followed by cleanup with RNeasy Mini Kits (QIAGEN, Valencia, CA) according to manufacturer instructions. Equal amounts of RNA (micrograms) were pooled from each of the five fish per tank (three tanks or biological replicates) for every treatment. RNA was pooled from individual animals to reduce variability across replicates due to individual differences, allowing for analysis of gene expression changes that are representative of the population. A reference sample was created from RNA pooled from 15 vehicle control fish. RNA quality and quantity were assessed by agarose gel electrophoresis, spectrophotometric absorbency at 260/280 nm, and bioanalyzer trace (Bioanalyzer 2100; Agilent, Palo Alto, CA).

### Peroxisomal β-oxidation and catalase activity

The β-oxidation of palmitoyl coenzyme A (CoA) was measured by the spectrophotometric method of Mitchell et al. (1985) in peroxisomal fractions prepared by differential centrifugation, as described previously (Orner et al. 1995). Enzyme activity was measured at 340 nm (20°C) and expressed as amount of NAD^+^ (nicotinamide adenine dinucleotide) reduced per minute per milligram of protein. Catalase activity was measured spectrophotometrically by the decay of hydrogen peroxide, as described previously (Aebi 1984; Orner et al. 1995). Enzyme activity was measured at 240 nm (20°C) and expressed as specific activity (micromoles per minute per milligram of protein). Protein was quantified by the method of Lowry et al. (1951).

### Serum VTG and E_2_

VTG was quantified in trout serum by enzyme-linked immunosorbent assay (ELISA), as previously described (Tilton et al. 2006). Protein concentrations were determined by the method of Lowry et al. (1951). VTG concentrations were determined by optical density on a SpectraMax 190 plate reader (Molecular Devices, Sunnyvale, CA) based on comparison with a trout VTG standard curve with a detection limit for this assay of 6.25 ng/mL. Serum VTG was also measured by Western blot using the same antibody to capture images from representative samples (Oganesian et al. 1999). E_2_ was quantified in trout serum by enzyme immunosorbent assay according to the manufacturer protocol (Cayman Chemical, Ann Arbor, MI).

### Microarray hybridization and analysis

Rainbow trout 70-mer oligonucleotide arrays (OSUrbt, version 2.0) containing 1,672 elements, representing approximately 1,400 genes, were created at Oregon State University. Microarray construction and quality control have been described previously (Tilton et al. 2005). Hybridizations were performed with the Genisphere Array 350 kit and instructions (Genisphere, Hatfield, PA) using standard reference design with dye swapping as described (Tilton et al. 2007). Data were background-subtracted and normalized by locally weighted scatterplot smoothing (LOWESS), which is recommended for two-color experiments to eliminate dye-related artifacts and produce ratios that are not affected by signal intensity values. Stringent criteria were used to filter for genes that were regulated at least 1.8-fold consistently in all features from biological replicates and had a *p-*value < 0.05 by Welch’s *t*-test (GeneSpring version 6; Silicon Genetics, Redwood City, CA). Genes that met these criteria were minimally categorized based on function using Gene Ontology (Gene Ontology Consortium 2008) and OMIM [National Center for Biotechnology Information (NCBI) 2008c] databases for putative homolog descriptions. Hierarchical clustering of gene expression profiles was performed in GeneSpring, and comparisons of microarray and real-time polymerase chain reaction (PCR) gene regulation were performed with GraphPad Prism (GraphPad Software, San Diego, CA).

### Real-time quantitative reverse transcription (qRT)-PCR

To assess the authenticity of results from the microarray analyses, we also analyzed mRNAs for select genes using real-time RT-PCR. Total RNA was isolated as described above and was treated with DNase (Invitrogen) according to manufacturer’s protocol. cDNA was synthesized from 2 μg RNA with an oligo (dT)_18_ primer using SuperScript II (Invitrogen) following the manufacturer’s instructions, with a final volume of 100 μL. Synthesized cDNAs (1 μL) were used as templates for amplification of specific gene products in total volumes of 20 μL containing 1X SYBR Green master mix (DyNAmo qPCR kit; Finnzymes, Espoo, Finland) and 0.3 μM of each primer. Primer sequences were as follows: 5′-GAGTTTGGGCAGGTGGTG-3′ and 5′-TGGTGCGGTTTGGTAGGT-3′ for cytochrome P450 family 1A [*CYP1A*; Dana-Farber Cancer Institute (DFCI 2008) Gene Index ID TC63282]; 5′-GTGTCAACTCTAATCTAGTGCCC-3′ and 5′-CCGTCCCTGATTGAAGTGAC-3′ for *CYP2K5* (TC95312); 5′-TAAAAGTTGCACAAGTTTCC-3′ and 5′-AAAGGTCCGTTCTGATCGTC-3′ for cathepsin D (*CTSD* (TC128395); 5′-AGCTCCTGCTCCTGCTCT-3′ and 5′-GGAATGGGCATCTGGTCT-3′ for estrogen receptor (ER)- α (*ESR1;* TC94766); 5′-CCAACCAAACGCTACCGAAC-3′ and 5′-CCAGATTCCATCTCACCTT-3′ for glyceraldehyde-3-phosphate dehydrogenase (*GAPDH*; TC94858); and 5′-TTGCCTTTGCCAACATCGAC-3′ and 5′-CGGACATTGACGTATGCTTT-3′ for *VTG* (TC47576). PCR was performed using a DNA Engine Cycler and Opticon 2 Detector (MJ Research, Waltham, MA). DNA amplification was quantified (picograms) from the C(T) value based on standard curves to ensure that quantification was within a linear range. Standards were created from gel-purified PCR products (QIAX II; QIAGEN) for each primer set after quantification with the PicoGreen dsDNA Quantification Kit (Molecular Probes, Eugene, OR) and serial dilutions ranging from 0.25 to 100 ng DNA. All signals were normalized against GAPDH, and ratios were calculated for treated samples compared with control. Because expression of GAPDH was not altered by treatment based on either microarray analysis or RT-PCR, we found it to be an appropriate housekeeping gene for normalization in this study.

## Results

### Tumor study

**Figure 1:**
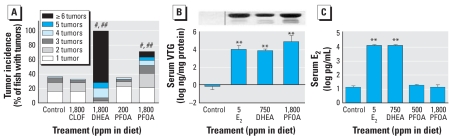
Effects of test compounds on tumor incidence, serum VTG, and serum E_2_ compared with DMSO vehicle control. (*A*) Tumor incidence and multiplicity after exposure to CLOF, DHEA, or PFOA in the diet for 6 months compared with control (postinitiation by immersion in 10 ppb AFB_1_ (each treatment consisted of a single tank of 68–100 individuals). (*B*) Serum VTG in trout after exposure to E_2_, DHEA, and PFOA in the diet for 5 days as determined by Western blot (representative images shown) and ELISA (*n* = 4). (*C*) Serum E_2_ in trout after exposure to E_2_, DHEA, and PFOA in the diet for 14 days, as determined by enzyme immunosorbent assay. For (*B*) and (*C*), pools of blood plasma were obtained from five individual males in each replicate tank (*n* = 3). **p < 0.01 compared with control by one-way ANOVA with Dunnett’s multiple comparison test. ^#^*p* < 0.0001 for tumor incidence and ^##^*p* < 0.0001 for multiplicity compared with control, calculated by logistic regression analysis and Kruskal-Wallis test, respectively.

Exposure to experimental diets containing 1,800 ppm PFOA (equivalent to 50 mg/kg/day) or DHEA significantly (*p* < 0.0001) enhanced the incidence of liver tumors in AFB_1_-initiated trout above control animals (Figure 1). In addition, the multiplicity increased significantly (*p* < 0.0001) in both PFOA- and DHEA-fed trout (Figure 1A). Some animals fed promotional diets after initiation with AFB_1_ had more than six tumors per liver, compared with animals on the control diet, most of which had 1–2 tumors per liver (in tumor-bearing animals). In contrast, postinitiation feeding with CLOF resulted in no increase in either tumor incidence or multiplicity. The historical spontaneous liver tumor incidence in 9-month-old trout fed control diet is 0.1%. Consequently, no tumors were observed in noninitiated animals fed control, CLOF, or PFOA diets. However, consumption of 1,800 ppm DHEA in the diet for 6 months resulted in 20% tumor incidence in noninitiated animals, which is consistent with previous observations in our laboratory that DHEA acts as a complete hepatocarcinogen in trout (Table 1) (Orner et al. 1995). Histologic examination of liver tumors confirmed previous findings from our laboratory that mixed carcinoma is the predominant tumor type in AFB_1_-initiated trout (Table 1) (Oganesian et al. 1999). Tumor type remained consistent among all treatments except in animals fed CLOF post-initiation, which resulted in a shift in predominant tumor type to cholangiocellular carcinoma. Both DHEA and PFOA also produced hepatomegaly, as measured by liver somatic index (LSI; liver weight ÷ body weight × 100). Trout fed 1,800 ppm PFOA or DHEA for either 2 or 10 weeks had a significantly greater LSI compared with controls (*p* < 0.05) (Table 2).

Despite significant tumor promotion and hepatomegaly in trout liver after PFOA treatment, both of which are characteristic of PPs, typical enzymatic measurements of liver peroxisomal activity were not elevated by PFOA. Analysis of hepatic peroxisomal palmitoyl CoA oxidase activity in trout fed PPs for 6 months provided no evidence that CLOF, DHEA, or PFOA induced peroxisome enzymatic activities (Table 2). In fact, significant reduction in β-oxidation was observed for 1,800 ppm PFOA and DHEA (*p* < 0.01). Similarly, β-oxidation and catalase activity were not increased in liver peroxisomes and liver homogenates of animals from the microarray experiment after 14-day exposure to experimental diets (Table 2). Ultra-structural examination of treated livers revealed no evidence of increased size or number of peroxisomes (data not shown). Because DHEA was previously found to induce hepatic VTG, a glucolipoprotein egg yolk precursor produced in response to estrogen (Orner et al. 1996), animals exposed to PFOA were examined for induction of this estrogenic marker. VTG was subsequently detected in serum of animals treated with 1,800 ppm PFOA or 750 ppm DHEA in the diet for 5 days compared with E_2_, which was included as a positive control, indicating that these compounds may induce an estrogenic response in trout (Figure 1B).

### Gene expression analysis

**Figure 2:**
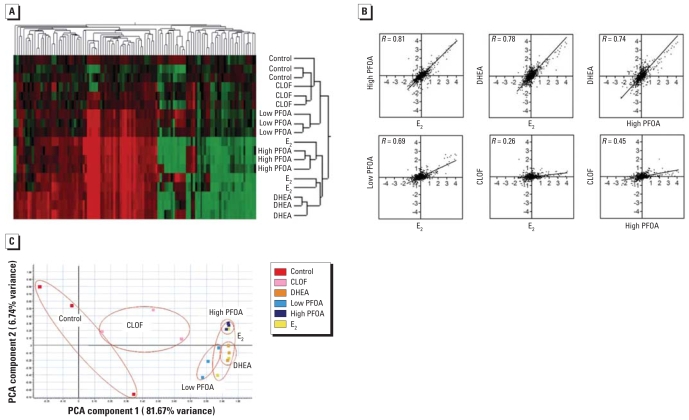
Hepatic gene expression analysis after dietary exposure to 0.1% DMSO (control), 500 or 1,800 ppm PFOA, 750 ppm DHEA, 1,800 ppm CLOF, or 5 ppm E_2_ for 14 days. PCA, principal components analysis. (*A*) Bidirectional hierarchical clustering of hepatic gene expression in trout by Euclidean distance. Results are shown as fold change (log_2_) of control of dye-swapped slides for biological replicates (*n* = three per treatment). Heatmap reflects expression profiles for genes differentially regulated 1.8-fold up or down (*p* < 0.05) in at least one treatment group. Red indicates up-regulation; green, down-regulation; black, unchanged expression; and grey, missing values. (*B*) Pairwise correlations of hepatic gene profiles; values are fold change (log_2_) compared with control and were plotted to generate Pearson correlation coefficients (*R*) among the treatments (*p* < 0.0001). Lines indicate least-squares linear regression. (*C*) PCA on condition. Symbols represent biological replicates (*n* = 3), and ovals indicate overlap among E_2_, PFOA, and DHEA treatments.

To evaluate the mechanism of tumor enhancement by PFOA, the OSUrbt, version 2.0, array was used to characterize transcriptional profiles in liver samples from animals treated with 1,800 ppm CLOF, 750 ppm DHEA, 500 (low) and 1,800 (high) ppm PFOA, or 5 ppm E_2_ in the diet compared with control animals. Supplemental raw data files are available online through Gene Expression Omnibus accession no. GSE7837 (NCBI 2008b). Array hybridizations were performed with a common reference sample using dye swapping, and final fold-change values were calculated as a ratio to control animals. Bidirectional hierarchical clustering of genes differentially regulated in at least one treatment group (Figure 2A) indicated similarities in the transcriptional profiles for E_2_, PFOA, and DHEA treatments. This is supported by pairwise analysis of all 1,672 features on the array using Pearson correlation, which demonstrated strong correlations in gene patterns between E_2_ and low PFOA, high PFOA, and DHEA [*R* = 0.69, 0.81, and 0.79 (two-tailed *p* < 0.0001), respectively] (Figure 2B). In contrast, gene profiles from CLOF had a low correlation with E_2_ (*R* = 0.26) and high PFOA (*R* = 0.45). Genes were considered differentially expressed if their mRNA levels changed ≥ or ≤ 1.8-fold compared with controls (*p* < 0.05) among biological replicates (Table 3). Transcriptional profiles for PFOA were typical of an estrogenic response in trout liver (Tilton et al. 2006) and strongly overlapped with the profile for E_2_ as determined by principal component analysis applied on condition (Figure 2C). Transcripts encoding vitellogenic liver proteins were the most sensitive markers for the estrogenic response in trout; however, a number of genes important for cell proliferation, protein transport, immune function, and metabolism were also differentially regulated by PFOA, DHEA, and E_2_ treatments. Interestingly, PFOA did not regulate many genes in common with CLOF, another PPAR agonist. *CYP2K5* was significantly up-regulated by both CLOF and PFOA and was down-regulated by E_2_ and DHEA. Microarray transcripts for carnitine palmitoyl-transferase II, acetyl-CoA acetyltransferase 2, and catalase, as measures of β-oxidation and hydrogen peroxide generation, were not differentially regulated (up or down) by treatment with the PPs or E_2_, supporting the lack of treatment-related elevation of peroxisomal enzyme activities observed in trout (Table 3). The ability of PFOA and DHEA to alter serum E_2_ concentrations was explored as a possible mechanism for their estrogenic activity. Serum E_2_ was significantly elevated in animals fed 5 ppm E_2_ or 750 ppm DHEA in the diet for 14 days; however, serum E_2_ in PFOA-treated animals was not statistically different from control animals (Figure 1C), suggesting that the estrogenic effect of PFOA was not indirectly caused by altered serum E_2_ levels.

**Figure 3:**
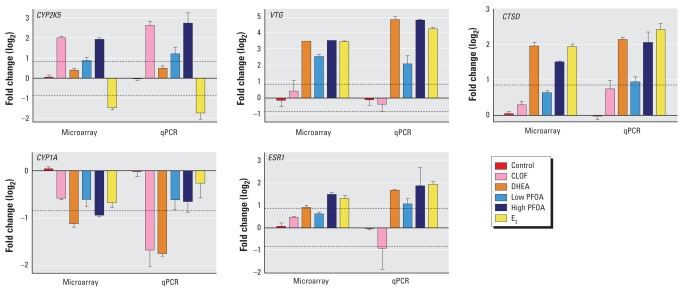
Comparison of gene expression of *CYP2K5*, *VTG*, *CTSD*, *CYP1A*, and *ESR1* measured by microarray and real-time qRT-PCR. Values are expressed as fold change (log_2_; mean ± SD) compared with vehicle control. Dashed lines indicate 1.8-fold change (± 0.847 log_2_).

Expression of select genes differentially increased or decreased by microarray analysis, including *CYP2K5*, *VTG*, *CYP1A*, *CTSD*, and *ESR1*, was confirmed by qRT-PCR using SYBR Green (Figure 3). Overall, we were able to confirm gene expression profiles measured by oligonucleotides microarray analysis using qRT-PCR. These data indicate that our strict criteria for determining differential gene regulation by array resulted in detection of meaningful changes that could be validated by other methods.

## Discussion

This study is the first report of tumor enhancement by PFOA in a model that is insensitive to peroxisome proliferation. Tumor promotion was not related to the function of PFOA as a PP or PPARα agonist but was phenotypically linked to estrogenic gene signatures in trout liver. The lack of tumor enhancement by CLOF is also a novel observation in trout and is supported by data indicating that another potent PP and PPARα agonist, Wy-14,643, does not enhance tumor incidence in trout after chronic exposure postinitiation (Carpenter H, personal communication). The tumor response to DHEA was similar to that published previously by our laboratory and confirms that DHEA is both a complete hepatocarcinogen and a tumor promoter in trout without inducing peroxisome proliferation (Orner et al. 1995). Chronic PFOA exposure causes liver tumors in rats and is associated with increased hepatomegaly, increased hepatic β-oxidation activity, and no change in hepatic cell proliferation (Biegel et al. 2001). These effects are typical of PPs in animals that are susceptible to peroxisome proliferation and further include increased size and number of peroxisomes and induction of peroxisomal and microsomal enzymes involved in β-oxidation and ω-oxidation of fatty acids (Moody et al. 1991). In the present study, PFOA exposure in trout resulted in hepatomegaly but no increase in the size or number of peroxisomes or in biochemical or transcriptional markers of peroxisome proliferation.

Compared with rodents, the effects observed in trout are more similar to those reported in nonhuman primates after oral exposure to the PFOA precursor ammonium perfluorooctanoate. In a study of male cynomolgus monkeys exposed to 3–30 mg/kg/day for 26 weeks, Butenhoff et al. (2002) observed increased liver weights but little or no histopathologic evidence of liver toxicity, changes in enzyme markers of peroxisome proliferation, or changes in serum hormone levels. The increase in monkey liver weights was attributed in part to hepatocellular hypertrophy and mitochondrial proliferation, as demonstrated by elevated succinate dehydrogenase activity. Succinate dehydrogenase was transcriptionally up-regulated in trout liver after exposure to PFOA, DHEA, and E_2_, all of which resulted in hepatomegaly in uninitiated animals. In addition, PFOA did not act as a complete carcinogen in either cynomolgus monkeys or trout after chronic exposure. One notable exception may be that cell proliferation, as measured by proliferating cell nuclear antigen (PCNA), was not elevated in cynomolgus liver at the end of 26 weeks, even though PFOA was detected in both liver and serum (Butenhoff et al. 2002). Although PCNA was not measured in the present study, previously it has been found to be induced in trout liver after 2-week exposure to DHEA, E_2_, and other compounds that result in estrogenic gene signatures (Orner et al. 1996; Tilton SC, unpublished data). The lack of PCNA as a proliferative marker in monkeys may indicate some differences in the mechanism for liver hypertrophy between trout and primates and should be further explored.

PPs are thought to cause cancer through the metabolism of long-chain fatty acids by the peroxisomal β-oxidation system, which generates hydrogen peroxide and can result in DNA damage. However, we found no evidence of oxidative stress after exposure to PPs in trout. Catalase was not increased by any PP treatment in this experiment. Glutathione peroxidase, another measure of oxidative stress, was only up-regulated by treatments that resulted in an estrogenic response and thus was not specific to the PP class of compounds. Interestingly, there were few transcriptional changes by PFOA in common with the other PPARα agonist, CLOF, and independent of E_2_. These changes included up-regulation of CYP2K5, prostaglandin D synthase, and to a lesser extent, carbonyl reductase. Although the function of CYP2K5 in trout is unknown, this gene was regulated by PPARα agonists and E_2_ in a manner that has been reported previously for CYP2K1, lauric acid ω-hydroxylase, which is known to be mediated by PPARα and shares approximately 80% sequence identity with *CYP2K5* (Buhler et al. 2000; Kennedy et al. 2004). However, *CYP2K1* itself was not transcriptionally regulated by any treatments in the present study, suggesting that these enzymes have different activities in trout liver. Both prostaglandin D synthase and carbonyl reductase are involved in the signaling and metabolism of prostainoids, a pathway that has been associated with development of hepatocellular carcinoma involving cyclo-oxygenase-2 (Wu 2006). Therefore, PFOA exposure resulted in some transcriptional responses that were specific for PPARα agonists and distinct from E_2_; however, they were not correlated with tumor enhancement by DHEA and PFOA.

Previously, we found that rainbow trout are very sensitive to promotion of hepatocarcinogenesis by E_2_ and other estrogenic compounds (Nunez et al. 1989; Tilton et al. 2007). The mechanism by which PFOA induces an estrogenic response in trout liver is currently unknown; however, some links between PPs and estrogen-mediated carcinogenesis have been reported. For example, fibrates can stimulate the esterification of E_2_ with fatty acids, which promote the storage and prolonged release of E_2_ from fatty tissue, enhancing cell proliferation in mammary gland (Yu et al. 2001). However, this mechanism was not relevant to PPs in trout liver because CLOF did not result in an estrogenic response. In other studies, the ability of PFOA to increase serum E_2_ by hepatic aromatase was correlated with the occurrence of rat Leydig cell tumors in the absence of peroxisome proliferation (Biegel et al. 2001). This mechanism is likely also irrelevant for PFOA in trout because it did not elevate serum E_2_. In comparison, serum E_2_ was increased after DHEA treatment, suggesting that DHEA induces an estrogenic response via indirect conversion of E_2_ by aromatase, as described previously (Benninghoff and Williams 2008). These data demonstrate that PFOA enhances tumorigenesis through an estrogenic mechanism unique among the PPs investigated in this study.

The potential for PFOA and other structurally similar perfluorinated chemicals to promote hepatocarcinogenesis via direct interactions with the trout hepatic ER is the subject of ongoing studies in our laboratory. Evidence for activation of ER by PFOA, perfluorooctanyl sulfonate, and several fluorotelomer alcohols is supported by data that the ER antagonist tamoxifen inhibits their estrogenic activity in primary tilapia hepatocytes *in vitro* (Liu et al. 2007). Up-regulation of ER-α and ER-β expression was also reported in human MCF-7 cells and Chinese rare minnow, respectively, after exposure to PFOA (Maras et al. 2006; Wei et al. 2007). However, PFOA does not transactivate human ER-α or ER-β or Japanese medaka ER-α in yeast two-hybrid assays, suggesting that either species selectivity in ER binding or indirect activation of ER may be occurring (Ishibashi et al. 2007, 2008). Cross-talk between nuclear receptors PPARγ, PPARα, and ER has been described in mouse uterus (Gunin et al. 2004), and PFOA activation of other nuclear receptors, including constitutive androstane receptor and pregnenolone X receptor, was reported in PPARα-null mice, possibly leading to hepatomegaly in these animals (Anderson et al. 2007). Therefore, although the majority of the hepatic effects of PFOA are mediated by PPARα in PP-sensitive models, interaction of PFOA with other nuclear receptors, including ER, may be functionally significant in liver of PP-insensitive models or in other target organs and merits further investigation.

It is clear that the specific mechanism for PFOA estrogenicity will be important in extrapolating these data across species; however, our current study provides evidence that animals lacking peroxisome proliferation activity are rather insensitive to carcinogenesis by PFOA. Only 50 mg/kg/day (1,800 ppm) PFOA resulted in tumor enhancement, indicating that chronic exposure to high levels of PFOA are necessary to promote hepatocarcinogenesis in trout, which are sensitive to liver tumor promotion by estrogens. These doses are typical of those used in rodent cancer studies with PPARα agonists but higher than the low part-per-billion PFOA concentrations measured in human and environmental samples (Calafat et al. 2007; Houde et al. 2006). Although our study did not identify a threshold for the estrogenic effects of PFOA in trout liver, we observed only 26% overlap of gene signatures between 5 ppm E_2_ and 500 ppm (5 mg/kg/day) PFOA compared with 83% at 1,800 ppm PFOA. These data demonstrate that PFOA is a weak estrogen in trout, similar to observations from other models in which PFOA weakly induced ER gene targets and also antagonized the estrogenic activity of E_2_ (Liu et al. 2007; Maras et al. 2006). Further studies are necessary to evaluate the potential for PFOA-mediated carcinogenesis in other PP-insensitive species, and in light of the mechanism identified in this study, the consequences of hormone-related effects by PFOA should be also considered in other tissues, models, and sensitive life stages.

## Conclusions

We report the novel findings that PFOA can enhance hepatocarcinogenesis postinitiation in rainbow trout, a model that is insensitive to peroxisome proliferation. Tumorigenesis was not related to the function of PFOA as a PP or PPARα agonist and was not observed after treatment with CLOF. Rather, tumor outcome was phenotypically linked to estrogenic gene signatures in trout liver after molecular profiling, which showed excellent correlation with E_2_ and is a unique mechanism action identified for PFOA in carcinogenesis.

## Figures and Tables

**Table 1:** Effects of PPs on tumor type.

Treatment^a^ (initiation/promotion)	Percent tumor incidence	Tumor class (%)						
		MC	HCC	CCC	MA	HCA	Ch	BF
None/none	0	0	0	0	0	0	0	0
None/CLOF	0	0	0	0	0	0	0	0
None/DHEA	20*	67	25	0	4	4	0	0
None/low PFOA	0	0	0	0	0	0	0	0
None/high PFOA	0	0	0	0	0	0	0	0
AFB_1_/none	36	54	10	10	5	3	5	12
AFB_1_/CLOF	32	15	0	74	0	2	7	2
AFB_1_/DHEA	100*	72	25	< 1	0	1	1	< 1
AFB_1_/low PFOA	34	50	11	16	5	0	14	5
AFB_1_/high PFOA	71*	37	46	1	2	8	1	5

Abbreviations: BF, basophilic foci; CCC, cholangiocellular carcinoma; Ch, cholangioma; HCA, hepatocellular adenoma; HCC, hepatocellular carcinoma; MA, mixed adenoma; MC, mixed carcinoma.

a Animals were initiated with 10 ppb AFB_1_ as fry and fed control diet for 3 months, followed by 6-month exposure to control, 1,800 ppm CLOF, 1,800 ppm DHEA, or 200 or 1,800 ppm PFOA in the diet postinitiation (promotion).

* *p* < 0.05 compared with control animals (within initiator group) by logistic regression analysis.

**Table 2:** Effects of PPs on liver weight and enzyme markers.

Treatment^a^	LSI^b^	Palmitoyl CoA β-oxidation^c^	Catalase^d^
Tumor study			
Control	1.572 ± 0.370	36.8 ± 5.4	
1,800 ppm CLOF	1.381 ± 0.270	27.1 ± 3.4	
1,800 ppm DHEA	4.889 ± 1.041***	10.0 ± 4.6**	
200 ppm PFOA	1.631 ± 0.259	23.8 ± 3.6	
1,800 ppm PFOA	2.440 ± 0.210***	15.2 ± 1.4**	
Microarray study			
Control	1.172 ± 0.039	63.5 ± 11.9	1033.0 ± 120.3
1,800 ppm CLOF	1.429 ± 0.095	52.3 ± 8.9	1105.0 ± 64.1
750 ppm DHEA	1.963 ± 0.101**	39.5 ± 2.5	633.1 ± 52.3*
500 ppm PFOA	1.513 ± 0.061*	58.6 ± 12.6	883.1 ± 28.9
1,800 ppm PFOA	1.543 ± 0.129*	26.5 ± 12.3	790.6 ± 104.5
5 ppm E_2_	1.930 ± 0.262**	23.3 ± 7.0	656.2 ± 45.5*

a Tumor study animals (*n* = 4; pool of three animals each) were initiated with 10 ppb AFB_1_ as fry and fed control diet for 3 months, followed by 10-week (LSI) or 6-month (β-oxidation) exposure to indicated treatments in the diet postinitiation (promotion). Microarray study animals (*n* = 3) were exposed as juveniles to indicated treatments in the diet for 14 days.

b Mean ± SD.

c Nanomoles of activity per minute per milligram protein (mean ± SE).

d Micromoles of activity per minute per milligram protein (mean ± SE).

* *p* < 0.05,

** *p* < 0.01, and

*** *p* < 0.001 compared with respective control values by one-way ANOVA with Dunnett’s multiple comparison test.

**Table 3:** Select genes differentially regulated in trout liver.

			Treatment (ppm)					
Array ID	DFCI ID^a^	Gene name (accession number, species)^b^	Control	CLOF 1,800	DHEA 750	PFOA 500	PFOA 1,800	E_2_ 5
Liver-specific proteins (vitellogenesis)								
OmyOSU208	TC94769	Vitellogenin precursor (Q92093; *O. mykiss*)	−0.06	0.52	3.90*	2.68*	4.15*	3.94*
OmyOSU203	TC47576	Vitellogenin precursor (X92804; *O. mykiss*)	−0.12	0.41	3.49*	2.54*	3.53*	3.47*
OmyOSU222	TC47576	Vitellogenin precursor (X92804; *O. mykiss*)	−0.19	0.29	3.22*	2.35*	3.43*	3.35*
OmyOSU248	TC47577	Vitellogenin (X92804; *O. mykiss*)	−0.27	0.23	2.98*	2.29*	3.12*	3.08*
OmyOSU1552	TC55460	Vitelline envelope protein gamma (AF231708; *O. mykiss*)	0.05	0.63	2.78*	2.63*	2.96*	2.84*
OmyOSU1542	TC85700	Zona radiata structural protein (AF407574; *O. mykiss*)	−0.04	0.60	2.72*	2.20*	2.63*	2.72*
Cell proliferation (signal transduction, growth factors, and apoptosis)								
OmyOSU212	TC70106	TATA-binding protein (AY168633; *Danio rerio*)	0.07	0.14	2.54*	1.36*	3.40*	3.18*
OmyOSU244	NP543968	Estrogen receptor-β(*ESR2*; AJ289883; *O. mykiss*)	0.02	0.21	1.92*	1.34*	2.13*	2.13*
OmyOSU151	TC88754	Estrogen receptor-α (*ESR1*; M31559; *O. mykiss*)	0.06	0.43	1.55*	0.72	2.08*	1.89*
OmyOSU150	TC94766	Estrogen receptor-α (*ESR1*; P16058; *O. mykiss*)	0.07	0.47	0.88*	0.59	1.46*	1.27*
OmyOSU673	TC94766	Estrogen receptor-α (*ESR1*; P16058; *O. mykiss*)	0.01	0.79	0.81	0.48	1.37*	1.00*
OmyOSU800	TC72880	Nonreceptor tyrosine kinase 2 (*TYK2*; AF173032; *Mus musculus*)	−0.03	0.07	1.07*	0.54	1.49*	1.44*
OmyOSU915	TC78497	Cysteine-rich with EGF-like domains 1 (CR751234; *D. rerio*)	0.08	−0.06	1.01*	0.20	0.91*	1.01*
OmyOSU242	TC108885	Growth hormone (P87487; *Oncorhynchus kisutch*)	0.01	0.05	1.03*	0.33	1.30*	1.23*
OmyOSU1427	TC100257	Reticulon RTN9-A2 (Q6IEI9; *O. mykiss*)	−0.03	0.18	1.95*	0.57	2.01*	1.80*
OmyOSU1428	TC103467	Reticulon RTN9-A1 (Q6IEJ0; *O. mykiss*)	0.01	0.26	1.72*	0.52	1.44*	1.57*
OmyOSU1588	TC96803	Viperin-like protein (Q5EEZ3; *Channa argus*)	0.10	0.08	1.17*	0.58	1.76*	1.76*
OmyOSU1068	TC118990	*STAT 1-2* (O13132; *O. mykiss*)	0.13	0.11	1.10*	0.04	0.56	0.89*
OmyOSU1615	TC81096	*TM4SF5* tumor antigen (AF281357; *O. mykiss*)	−0.03	0.04	2.35*	0.28	0.66	0.95*
OmyOSU313	TC106070	Bone morphogenic protein 7 (S77477; *Gallus gallus*)	−0.01	−0.04	−1.59*	−0.10	−1.04*	−1.24*
Protein stability and transport								
OmyOSU139	TC128395	Cathepsin D (P87370; *O. mykiss*)	0.03	0.29	1.95*	0.64	1.50*	1.93*
OmyOSU1308	TC118879	Sec61 alpha form B (AF346601; *O. mykiss*)	−0.05	−0.15	1.74*	0.14	0.95*	1.51*
OmyOSU933	TC115491	Chaperone protein GP96 (tumor rejection antigen) (Q7T3L3; *D. rerio*)	0.03	−0.25	1.14*	0.24	0.84*	0.92*
OmyOSU1306	TC109072	Sec61 alpha form A (AF346600; *O. mykiss*)	0.29	0.14	1.03*	0.09	0.53	0.83
OmyOSU1458	TC106687	Tubulin alpha-3 chain (P68365; *Canis familiaris*)	−0.03	0.34	1.54*	0.26	0.37	1.00*
OmyOSU1460	TC98663	Beta tubulin (Q9DFT6; *Notothenia coriiceps*)	0.03	0.08	1.51*	0.01	0.06	0.89*
OmyOSU205	TC130693	Actin, cytoplasmic 1 (β-actin; O42161; *Salmo salar*)	0.15	0.14	1.14*	0.31	0.87*	0.79
OmyOSU1587	TC98530	Transgelin 2 (Q803W9; *D. rerio*)	0.07	0.14	0.94*	−0.16	−0.08	−0.10
Nucleic acid metabolism								
OmyOSU1518	TC131311	Similar to uridine phosphorylase 1 (XM_685152; *D. rerio*)	0.05	0.29	2.24*	1.55*	2.70*	3.01*
OmyOSU252	TC111966	Hypoxanthine phosphoribosyltransferase 1 (Q7ZV49; *D. rerio*)	0.13	0.24	1.65*	1.07*	2.29*	2.00*
OmyOSU1219	TC101340	Nucleoside diphosphate kinase (Q804Y0; *O. mykiss*)	−0.01	0.12	1.15*	0.24	0.52	0.64
Transcription and translation								
OmyOSU1439	TC107620	Translation factor sui1-like (Q2HYN5; *Ictalurus punctatus*)	0.01	0.45	0.97*	0.09	0.45	0.4
Immune function and acute-phase response								
OmyOSU1147	TC115303	Pentraxin precursor (P79899; *O. mykiss*)	0.07	0.22	−1.29*	−0.33	−0.93*	−1.37*
OmyOSU1236	CR375493	Apopolysialoglycoprotein precursor (J04051; *O. mykiss*)	0.01	−0.51	−0.93*	−0.37	−1.08*	−0.73
OmyOSU1512	TC87050	Apolipoprotein A1 (AB183290; *Fugu rubripes*)	−0.23	−0.96	−2.42*	−0.43	−2.34*	−0.51
OmyOSU878	CA367917	*LECT2* neutrophil chemotactic factor (AF363272; *O. mykiss*)	−0.14	−0.15	−0.87	−0.03	−1.94*	−0.84
OmyOSU1469	TC8260	Cathepsin S (AY950578; *Paralichthys olivaceus*)	0.06	−0.50	−0.72	−0.33	−1.21*	−0.85*
OmyOSU51	TC94755	*IL-8* receptor (Q90VZ2; *O. mykiss*)	−0.04	0.52	−1.01*	0.09	0.53	−0.90*
OmyOSU1669	NP814796	TNF receptor associated factor 2 (AJ548839; *O. mykiss*)	0.08	0.15	−0.37	−0.12	−1.22*	−0.96*
OmyOSU232	TC91273	Differentially regulated trout protein (AF281355; *O. mykiss*)	−0.34	−0.25	0.55	0.90	−1.58*	0.10
OmyOSU744	TC71412	Putative interlectin (AF281350; *O. mykiss*)	−0.06	−0.06	−1.09*	0.40	−0.31	−0.15
OmyOSU401	NP543817	Complement component C5 (AF349001; *O. mykiss*)	−0.06	0.05	−0.97*	−0.04	−0.29	−0.61
OmyOSU411	TC97418	Complement component C9 (Q4QZ25; *O. mykiss*)	0.04	0.03	−0.94*	−0.04	−0.37	−0.24
OmyOSU1648	TC113043	Haptoglobin 2 (Q9DFG0; *O. mykiss*)	−0.01	0.46	−1.59*	0.36	−0.46	0.00
OmyOSU821	TC113043	Haptoglobin 2 (Q9DFG0; *O. mykiss*)	−0.07	0.47	−1.79*	0.42	−0.52	−0.01
OmyOSU1564	TC100075	VHSV-induced protein-4 (Q8QGB4; *O. mykiss*)	0.00	0.65	3.75*	2.55*	3.98*	4.06*
OmyOSU1566	TC109185	VHSV-induced protein-6 (Q8QGB2; *O. mykiss*)	0.03	0.41	2.81*	1.81*	2.93*	3.27*
OmyOSU1580	TC104328	VHSV-induced protein (Q8QGA5; *O. mykiss*)	−0.08	0.05	0.74	0.36	1.06*	1.04*
OmyOSU1576	TC94860	VHSV-induced protein-7 (Q8QGB7; *O. mykiss*)	0.07	−0.09	1.16*	0.46	0.93*	0.88*
OmyOSU1586	TC96479	VHSV-induced protein (Q8QGA4; *O. mykiss*)	0.13	0.35	1.22*	1.25*	0.75	0.79
OmyOSU634	TC110053	VHSV-induced C-lectin-like protein (Q8QGA9; *O. mykiss*)	0.04	0.02	1.15*	0.35	0.74	0.75
OmyOSU1584	NP543665	VHSV-induced protein (AF483543; *O. mykiss*)	0.10	0.46	1.39*	1.35*	0.65	0.66
OmyOSU1192	TC94749	Tapasin short form (Q3SAV0; *O. mykiss*)	0.12	−0.17	0.95*	0.18	0.40	0.69
OmyOSU567	TC126430	Chemokine CK-1 (Q9W691; *O. mykiss*)	−0.02	0.01	2.52*	−0.15	−0.02	−0.14
Metabolism/homeostasis (drug, lipid, fatty acid, retinol)								
OmyOSU1389	TC95312	*CYP2K5* (AAC28309; *O. mykiss*)	0.05	2.02*	0.40	0.87*	1.95*	−1.47*
OmyOSU146	TC63282	*CYP1A* (AF059711; *O. mykiss*)	0.04	−0.58	−1.11*	−0.60	−0.94*	−0.68
OmyOSU147	NP543804	*CYP3A45* (AF267126; *O. mykiss*)	0.04	0.76	−1.00*	0.14	0.63	−0.93*
OmyOSU343	TC69983	Biotinidase fragment 2 (AF281333; *O. mykiss*)	−0.01	−0.07	−1.68*	−0.18	−1.14*	−1.52*
OmyOSU1131	TC105248	Arylamine *N*-acetyl transferase (Q3ZLG1; *Oreochromis mossambicus*)	0.05	0.50	−1.30*	−0.41	−0.67	−1.01*
OmyOSU153	TC89948	Liver fatty acid binding protein (AF281344; *O. mykiss*)	−0.10	0.33	1.67*	0.49	0.45	0.75
OmyOSU875	TC98662	Glucokinase (O93314; *O. mykiss*)	−0.03	0.60	1.87*	0.45	0.49	1.92*
OmyOSU873	TC98662	Glucokinase (O93314; *O. mykiss*)	0.06	0.58	1.65*	0.38	0.39	1.66*
OmyOSU249	TC106837	Succinate dehydrogenase, subunit A (Q7ZVF3; *D. rerio*)	−0.12	0.10	1.44*	0.98*	1.93*	1.92*
OmyOSU245	TC112292	Succinate dehydrogenase, subunit A (Q6TNQ9; *D. rerio*)	0.01	0.15	1.21*	0.76	1.75*	1.78*
OmyOSU1631	TC98907	Prostaglandin D synthase (Q9DFD7; *O. mykiss*)	−0.04	0.91*	1.16*	0.36	0.85*	0.25
OmyOSU847	TC94773	Carbonyl reductase/*HSD20BA* (Q9PT36; *O. mykiss*)	0.09	0.63	0.16	0.52	1.05*	0.04
OmyOSU547	TC111036	Carnitine *O*-palmitoyltransferase II (Q5U3U3; *D. rerio*)	−0.00	−0.17	−0.00	−0.15	−0.00	−0.08
OmyOSU327	TC96499	Acetyl-CoA acetyltransferase 2 (Q5SPA3; *D. rerio*)	0.00	−0.02	0.02	0.05	−0.18	−0.05
Redox regulation								
OmyOSU238	TC56389	Glutathione peroxidase 4 (AAO86704; *D. rerio*)	0.02	0.08	0.84*	0.55	1.16*	1.46*
OmyOSU37	TC101266	Catalase (AF170069; *D. rerio*)	−0.09	−0.19	−0.69	−0.43	−0.54	−0.69
Miscellaneous								
OmyOSU1034	TC111169	*SOX-LZ* (Q91215; *O. mykiss*)	0.02	0.97	0.92*	0.77	2.32*	1.15*
OmyOSU488	TC97895	DMRT4 protein (AF209097; *O. mykiss*)	0.02	0.43	0.74	0.31	1.41*	0.82
OmyOSU486	NP543963	DMRT2 protein (AF209096; *O. mykiss*)	0.00	−0.06	−0.99*	−0.20	−1.29*	−1.33*
OmyOSU243	TC110554	Ictacalcin (Q4JI13; *I. punctatus*)	0.01	0.17	1.86*	−0.02	0.17	−0.08
OmyOSU572	TC116478	COX6A mitochondrial precursor (O13085; *O. mykiss*)	0.06	0.14	−1.17*	−0.12	0.25	−0.15
OmyOSU544	TC105829	Small EDRK-rich factor 2 (P84101; *Homo sapiens*)	−0.02	−0.02	−1.30*	−0.04	−1.67*	−1.46*

Abbreviations: DMRT4, DM-related transcription factor 4; EGF, epidermal growth factor; IL, interleukin; TNF, tumor necrosis factor; VHSV, viral hemorrhagic septicemia virus. Values shown are average fold change values (log_2_) that represent background-corrected, LOWESS-normalized signal ratios.

a DFCI Gene Index ID number of the tentative consensus or singleton expressed sequence tag sequence corresponding to the OSUrbt, version 2, microarray feature.

b Data shown are the most significant BLASTX (NCBI 2008a); however, if an expressed sequence tag has no significant (E-value < 10^−6^) BLASTX hit, then the most significant BLASTN hit is shown. Genes have been categorized by function based on putative trout homolog using the Gene Ontology and OMIM databases (NCBI 2008b, 2008c). Animals were fed 0.1% DMSO vehicle (CON), 1,800 ppm CLOF, 750 ppm DHEA, or 500 or 1,800 ppm PFOA acid (low or high PFOA, respectively), or 5 ppm E_2_ in the diet for 14 days.

* *p* < 0.05 by Welch’s *t*-test for genes regulated at least 1.8-fold.

## References

[b1-ehp0116-001047] Abdellatif AG, Preat V, Taper HS, Robertfroid M (1991). The modulation of rat liver carcinogenesis by perfluorooctanoic acid, a peroxisome proliferator. Toxicol Appl Pharmacol.

[b2-ehp0116-001047] Aebi H (1984). Catalase *in vitro*. Method Enzymol.

[b3-ehp0116-001047] Anderson ME, Butenhoff JL, Change SC, Farrar DG, Kennedy GL, Lau C (2007). Perfluoroalkyl acids and related chemistries – toxicokinetics and modes of action. Toxicol Sci.

[b4-ehp0116-001047] Bailey GS, Williams DE, Hendricks JD (1996). Fish models for environmental carcinogenesis. Environ Health Perspect.

[b5-ehp0116-001047] Benninghoff AD, Williams DE (2008). Identification of a transcriptional fingerprint of estrogen exposure in rainbow trout liver. Toxicol Sci.

[b6-ehp0116-001047] Biegel LB, Hurtt ME, Frame SR, O’Connor JC, Cook JC (2001). Mechanisms of extrahepatic tumor induction by peroxisome proliferators in male CD rats. Toxicol Sci.

[b7-ehp0116-001047] Buhler DR, Miranda CL, Henderson MC, Yang YH, Lee SJ, Wang-Buhler JL (2000). Effects of 17β-estradiol and testosterone on hepatic mRNA/protein levels and catalytic activities of CYP2M1, CYP2K1 and CYP3A27 in rainbow trout (*Oncorhynchus mykiss*). Toxicol Appl Pharmacol.

[b8-ehp0116-001047] Butenhoff JL, Costa G, Elcombe C, Farrar D, Hanson K, Iwai H (2002). Toxicity of ammonium perfluorooctanoate in male cynomolgus monkeys after oral dosing for 6 months. Toxicol Sci.

[b9-ehp0116-001047] Butenhoff JL, Kennedy GL, Hinderliter PM, Lieder PH, Jung R, Jansen KJ (2004). Pharmacokinetics of perfluorooctanoate in cynomolgus monkeys. Toxicol Sci.

[b10-ehp0116-001047] Calafat AM, Kuklenyik Z, Reidy JA, Caudill SP, Tully JS, Needham LL (2007). Serum concentrations of 11 polyfluoro-alkyl compounds in the U.S. population: data from the national health and nutrition examination survey (NHANES) 1999–2000. Environ Sci Technol.

[b11-ehp0116-001047] Fruchart JC, Brewer HB, Leitersdorf E (1998). Consensus for the use of fibrates in the treatment of dyslipoproteinemia and coronary heart disease. Fibrate Consensus Group. Am J Cardiol.

[b12-ehp0116-001047] DFCI (Dana Farber Cancer Institute) (2008). The Gene Index Project.

[b13-ehp0116-001047] Gene Ontology Consortium (2008). The Gene Ontology.

[b14-ehp0116-001047] Gunin AG, Bitter AD, Demakov AB, Vasilieva EN (2004). Effects of peroxisome proliferator activated receptors-α and –γ agonists on estradiol-induced proliferation and hyperplasia formation in the mouse uterus. J Endocrinol.

[b15-ehp0116-001047] Guruge KS, Yeung LWY, Yamanaka N, Miyazaki S, Lam PKS, Giesy JP (2006). Gene expression profiles in rat liver treated with perfluorooctanoic acid (PFOA). Toxicol Sci.

[b16-ehp0116-001047] Hendricks JD, Meyers TR, Shelton DW (1984). Histological progression of hepatic neoplasia in rainbow trout (*Salmo gairdneri*). Monogr Natl Cancer Inst.

[b17-ehp0116-001047] Houde M, Martin JW, Letcher RJ, Solomon KR, Muir DCG (2006). Biological monitoring of polyfluoroalkyl substances: a review. Environ Sci Technol.

[b18-ehp0116-001047] Ishibashi H, Ishida H, Matsuoka M, Tominaga N, Arizono K (2007). Estrogenic effects of fluorotelomer alcohols for human estrogen receptor isoforms α and β *in vitro*. Biol Pharm Bull.

[b19-ehp0116-001047] Ishibashi H, Yamauchi R, Matsuoka M, Kim JW, Hirano M, Yamaguchi A (2008). Fluorotelomer alcohols induce hepatic vitellogenin through activation of the estrogen receptor in male medaka (*Oryzias latipes*). Chemosphere.

[b20-ehp0116-001047] Kelly JD, Orner GA, Hendricks JD, Williams DE (1992). Dietary hydrogen peroxide enhances hepatocarcinogenesis in trout: correlation with 8-hydroxy-2′-deoxyguanosine levels in liver DNA. Carcinogenesis.

[b21-ehp0116-001047] Kennedy GL, Butenhoff JL, Olsen GW, O’Connor JC, Seacat AM, Perkins RG (2004). The toxicology of perfluorooctanoate. Crit Rev Toxicol.

[b22-ehp0116-001047] Lake BG, Evans JG, Gray TJB, Korosi SA, North CJ (1989). Comparative studies on nafenopin induced hepatic peroxisome proliferation in the rat, syrian hamster, guinea pig, and marmoset. Toxicol Appl Pharmacol.

[b23-ehp0116-001047] Lee BC, Hendricks JD, Bailey GS, Smith JE (1991). Toxicity of microtoxins in the feed of fish. Mycotoxins and Animal Feedstuff: Natural Occurrence, Toxicity and Control.

[b24-ehp0116-001047] Liu C, Du Y, Zhou B (2007). Evaluation of estrogenic activities and mechanism of action of perfluorinated chemicals determined by vitellogenin induction in primary cultured tilapia hepatocytes. Aquatic Toxicol.

[b25-ehp0116-001047] Lowry OH, Rosebrough NJ, Farr AL, Randall RJ (1951). Protein measurement with Folin phenol reagent. J Biol Chem.

[b26-ehp0116-001047] Maras M, Vanparys C, Muylie F, Robbens J, Berger U, Barber JL (2006). Estrogen-like properties of fluorotelomer alcohols as revealed by MCF-7 breast cancer cell proliferation. Environ Health Perspect.

[b27-ehp0116-001047] Mitchell AM, Lhuguenot JC, Bridges JW, Elcombe CR (1985). Identification of the proximate peroxisome proliferators derived from di(2-thylhexyl)phthalate. Toxicol Appl Pharmaol.

[b28-ehp0116-001047] Moody DE, Reddy JK, Lake BG, Popp JA, Reese DH (1991). Peroxisome proliferation and nongenotoxic carcinogenesis: commentary on a symposium. Fundam Appl Toxicol.

[b29-ehp0116-001047] NCBI (National Center for Biotechnology Information) (2008a). BLAST Home.

[b30-ehp0116-001047] NCBI (National Center for Biotechnology Information) (2008b). Gene Expression Omnibus.

[b31-ehp0116-001047] NCBI (National Center for Biotechnology Information) (2008c). Online Mendelian Inheritance in Man, OMIM.

[b32-ehp0116-001047] Nunez O, Hendricks JD, Arbogast DN, Fong AT, Lee BC, Bailey GS (1989). Promotion of aflatoxin B1 hepatocarcino-genesis in rainbow trout by 17β-estradiol. Aquat Toxicol.

[b33-ehp0116-001047] Oganesian A, Hendricks JD, Pereira CB, Orner GA, Bailey GS, Williams DE (1999). Potency of dietary indole-3-carbinol as a promoter of aflatoxin B_1_-initiated hepatocarcinogenesis: results from a 9000 animal tumor study. Carcinogenesis.

[b34-ehp0116-001047] Olsen GW, Burris JM, Ehresman DJ, Froehlich JW, Seacat AM, Butenhoff JL (2007). Half-life of serum elimination of perfluorooctanesulfonate, perfluorohexanesulfonate, and perfluorooctanoate in retired fluorochemical production workers. Environ Health Perspect.

[b35-ehp0116-001047] Orner GA, Donohoe RM, Hendricks JD, Curtis LR, Williams DE (1996). Comparison of the enhancing effects of dehydroepiandrosterone with the structural analog 16-fluoro-5-androsten-17-one on aflatoxin B_1_ hepatocarcinogenesis in rainbow trout. Fundam Appl Toxicol.

[b36-ehp0116-001047] Orner GA, Mathews C, Hendricks JD, Carpenter HM, Bailey GS, Williams DE (1995). Dehydroepiandrosterone is a complete hepatocarcinogen and potent tumor promoter in the absence of peroxisome proliferation in rainbow trout. Carcinogenesis.

[b37-ehp0116-001047] Palmer CAN, Hsu MH, Griffin KJ, Raucy JL, Johnson EF (1998). Peroxisome proliferators activated receptor-α expression in human liver. Mol Pharmacol.

[b38-ehp0116-001047] Peters JM, Hennuyer N, Staels B, Fruchart JC, Fievet C, Gonzalez FJ (1997). Alterations in lipoprotein metabolism in peroxisome-proliferator-activated receptor alpha-deficient mice. J Biol Chem.

[b39-ehp0116-001047] Prevedouros K, Cousins IT, Buck RC, Korzeniowski SH (2006). Sources, fate and transport of perfluorocarboxylates. Environ Sci Technol.

[b40-ehp0116-001047] Reddy JK, Qureshi SA (1979). Tumorigenicity of the hypolipidaemic peroxisome proliferators ethyl-alpha-*p*-chlorophenoxyi-sobutyrate (clofibrate) in rats. Br J Cancer.

[b41-ehp0116-001047] Reddy JK, Rao MS (1989). Oxidative DNA damage caused by persistent peroxisome proliferation: its role in hepatocarcinogenesis. Mutat Res.

[b42-ehp0116-001047] Sohlenius AK, Lundgren B, DePierre JW (1992). Perfluorooctanoic acid has persistent effects on peroxisome proliferation and related parameters in mouse liver. J Biochem Toxicol.

[b43-ehp0116-001047] Tilton SC, Gerwick LG, Hendricks JD, Rosato C, Corley-Smith G, Givan SA (2005). Use of a rainbow trout oligonucleotide microarray to examine transcriptional patterns in aflatoxin B_1_-induced hepatocellular carcinoma compared to adjacent liver. Toxicol Sci.

[b44-ehp0116-001047] Tilton SC, Givan SA, Pereira CB, Bailey GS, Williams DE (2006). Toxicogenomic profiling of the hepatic tumor promoters indole-3-carbinol, 17β-estradiol and β-naphthoflavone in rainbow trout. Toxicol Sci.

[b45-ehp0116-001047] Tilton SC, Hendricks JD, Orner GA, Pereira CB, Bailey GS, Williams DE (2007). Gene expression analysis during tumor enhancement by the dietary phytochemical, 3,3′-diindolyl-methane, in rainbow trout. Carcinogenesis.

[b46-ehp0116-001047] U.S. Environmental Protection Agency (2006). SAB Review of the EPA Draft Risk Assessment of Potential Human Health Effects Associated with PFOA and Its Salts.

[b47-ehp0116-001047] Vanden Heuvel JP, Kuslikis BI, Van Rafelghem MJ, Peterson RE (1991). Tissue distribution, metabolism and elimination of perfluorooctanoic acid in male and female rats. J Biochem Toxicol.

[b48-ehp0116-001047] Webb SJ, Geoghegan TE, Prough RA, Micheal Miller KK (2006). The biological actions of dehydroepiandrosterone involves multiple receptors. Drug Metab Rev.

[b49-ehp0116-001047] Wei Y, Dai J, Liu M, Wang J, Xu M, Zha J (2007). Estrogen-like properties of perfluorooctanoic acid as revealed by expressing hepatic estrogen-responsive genes in rare minnows (*Gobiocypris rarus*). Environ Toxicol Chem.

[b50-ehp0116-001047] Wu T (2006). Cyclooxygenase-2 in hepatocellular carcinoma. Cancer Treat Rev.

[b51-ehp0116-001047] Yang Q, Xie Y, Alexson SHE, Nelson BD, DePierre JW (2002). Involvement of the peroxisome proliferators-activated receptor alpha in the immunomodulation caused by peroxisome proliferators in mice. Biochem Pharmacol.

[b52-ehp0116-001047] Yu S, Zhu BT, Conney AH (2001). Stimulatory effect of clofibrate and gemfibrozil administration on the formation of fatty acid esters of estradiol by rat liver microsomes. J Pharmacol Exp Ther.

